# Treatment of Human Intraosseous Periodontal Defects Using Recombinant Human Bone Morphogenetic Protein-2: A Randomized Controlled Clinical Trial

**DOI:** 10.7759/cureus.40395

**Published:** 2023-06-14

**Authors:** Shashank Garg, Radhika Kapoor, Prashant Tyagi, Amit Wadhawan

**Affiliations:** 1 Department of Periodontology, Shree Bankey Bihari Dental College and Research Centre, Ghaziabad, IND

**Keywords:** periodontitis, open flap debridement, alloplast, intraosseous defects, rhbmp

## Abstract

Aim

The purpose of this research was to assess the clinical and radiographic outcomes of recombinant human bone morphogenetic protein-2 (rhBMP-2) for the treatment of intraosseous abnormalities after periodontal flap surgery.

Material and methods

Patients aged 35-55 years who had undergone periodontal treatment at Shree Bankey Bihari Dental College and Research Centre, Ghaziabad, and had a total of 14 intraosseous abnormalities were included in the research. Those in the control group had open flap debridement with alloplast, whereas those in the experimental group underwent the same procedure with the addition of rhBMP-2. Clinical indicators, such as plaque index (PI), gingival index, probing pocket depth (PPD), clinical attachment level, and radiographic defect fill, were collected at baseline at three months, six months, and nine months.

Results

The findings demonstrated that following periodontal treatment, both sets of patients had considerable improvements in their PI, gingival index, and PPD. The degree of relative connection improved significantly in both groups. When comparing the two groups radiographically, we saw that the test group had significantly better defect fill than the control group.

Conclusion

According to this research, there was a statistically significant decrease in PI, gingival index, PPD, clinical attachment level, and radiographic bone fill in patients who received rhBMP-2. Open flap debridement with rhBMP-2 and alloplastic bone grafts showed better reduction than open flap debridement with alloplastic bone grafts group in the radiographic defect fill.

## Introduction

The apical position of the base of the pocket in relation to the remnant alveolar crest (AC) is used to classify intraosseous abnormalities. Between 18% and 51% of people have at least one intraosseous defect, based on clinical and radiographic examinations. If intraosseous abnormalities are not corrected, they might worsen and eventually cause the loss of teeth [1]. Recombinant human bone morphogenetic protein-2 (rhBMP-2) technologies have been shown to improve the regeneration of alveolar bone and periodontal attachment in cases with medically induced and previously plaque- and calculus-exposed periodontal deficits. Periodontal regeneration after rhBMP-2 construct implantation has resulted in substantial regrowth of alveolar bone and cementum; however, there has been an inconsistent reformation of a functionally oriented periodontal ligament (PDL).

The primary goal of periodontal treatment is to provide a setting that promotes the continued health, comfort, and function of the patient’s dentition. Despite the availability of various therapies, dentists are always on the lookout for something that will be more reliable, less dependent on the skill of the practitioner, result in more rapid tissue regeneration, and be applicable to a wider range of periodontal abnormalities. Guided tissue regeneration (GTR), in which a membrane is used to prevent the proliferation of unwanted cells into the defect, can be used in conjunction with synthetic barrier membranes to promote appropriate progenitor cell populations at the wound site.

Growth factors, or morphogens, may be found in the body’s own cells and tissues, including bone, platelets, and many others. Differentiating stem cells into bone-forming cells (osteoinduction) and stimulating cell proliferation (mitogenic) and chemotaxis (cell recruitment) are the primary actions of growth factors and morphogens, respectively.

When present in the bone matrix, bone morphogenetic proteins (BMPs) exhibit osteogenic and osteoinductive capabilities that lead to the sequential creation of new bone. The BMPs are a group of over 20 closely related proteins that are classified as members of the transforming growth factor family. Devitalized and demineralized bone matrix, as described by Urist, Reddi, and Huggins, may stimulate osteogenesis in bone defects when implanted there over an extended length of time. Numerous studies have demonstrated that BMPs promote the regeneration of lost periodontal tissue in surgically created defects. rhBMP-2 treatment after periodontal reconstructive surgery in dogs resulted in substantial bone and cementum regeneration. Regeneration is enhanced when BMPs two and seven are applied to artificial periodontal lesions made surgically in rats and dogs. BMPs two and seven have been proven in in vitro investigations to stimulate osteoblastic and chondrocytic differentiation in addition to inducing bone and cementum production [2].

To evaluate the efficacy of rhBMP-2 in the management of periodontal intraosseous defects, its effects on clinical markers such as plaque index (PI), gingival index, probing pocket depth (PPD), clinical attachment level, and radiographic defect fill were documented.

## Materials and methods

The Department of Periodontology, Shree Bankey Bihari Dental College and Research Centre, Ghaziabad, recruited 14 participants aged 35-55 years for the research. This study was a randomized controlled trial that was carried out by tossing the coin and employed a split-mouth design. All 14 patients completed the study. The 28 intraosseous defects were selected and included: The control group included defects that were treated with open flap debridement (OFD) with bone graft, and the test group included defects that were treated with OFD with bone graft + rhBMP-2. The participants in this trial reported no side effects while using rhBMP-2, and the drug was shown to have great handling properties.

Clinical attachment level (CAL), probing pocket depth (PPD) measured from the gingival margin, and plaque index (PI) (Silness-Loe, 1964) were among the measures used.

The use of the paralleling method and holders, as well as the use of conventional radiographs, were among the many radiographic criteria evaluated. The distance between the alveolar crest (AC) and the base of the defect (BD), the distance between the cementoenamel junction (CEJ) and the BD, and the distance between the CEJ and the AC were all measured in millimeters.

Inclusion criteria were patients with an interproximal PPD ≥ 5 mm after phase I therapy (scaling and root planning) in asymptomatic teeth with intraosseous defects on an intraoral periapical radiograph (IOPAR). Exclusion criteria were patients with grade III stage three known systemic illness and periodontitis, which was stage III, under medications known to affect periodontal therapy, smoking or tobacco use, pregnancy or lactation, and an insufficient platelet count (< 200,000/mm3). Those with unacceptable oral hygiene and plaque index after re-evaluation of phase I therapy, those who had undergone periodontal therapy three months before the study, and those allergic to animal products were not included. Teeth with furcation defects, gingival recession, non-vitality, and/or mobility of at least grade II were not included.

**Figure 1:**
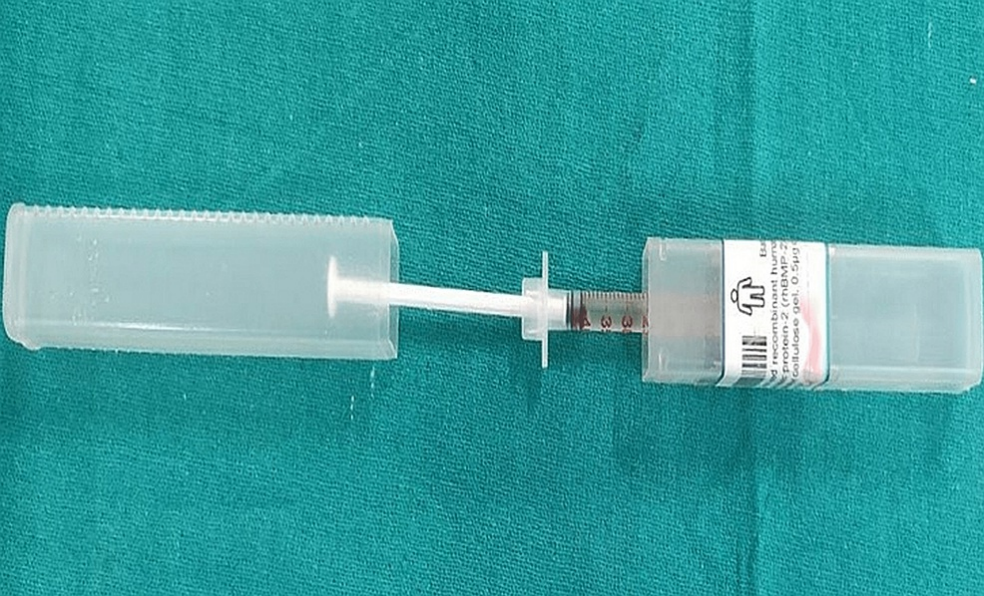
rhBMp-2 used rhBMP-2: recombinant human bone morphogenetic protein-2

**Figure 2:**
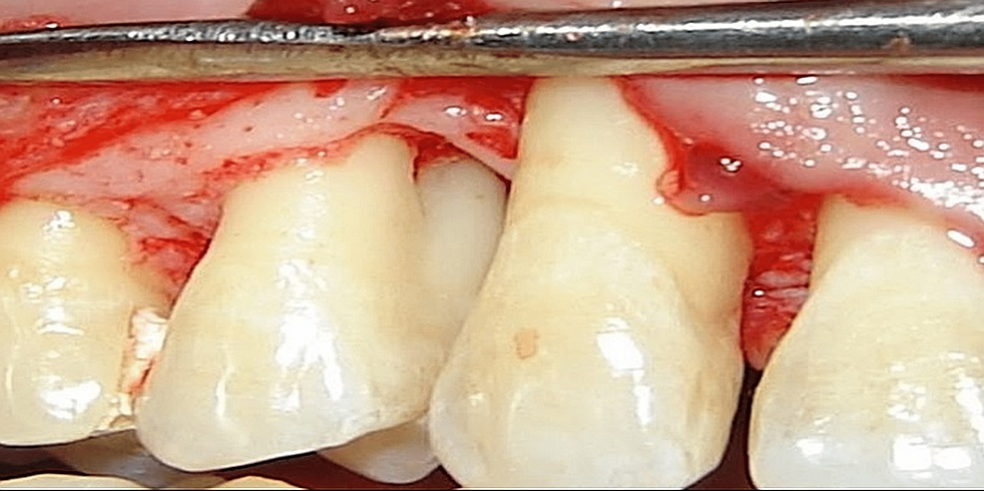
Flap reflection before placement of the rhBMP-2 along with bone graft rhBMP-2: recombinant human bone morphogenetic protein-2

The planned incision site was numbed with a local anesthetic. Sulcular incisions were used to raise buccal and lingual full-thickness (mucoperiosteal) flaps. After the mucoperiosteal flap was reflected, the granulation tissue surrounding the osseous defect was completely debrided. Both the control and test sites were reviewed thoroughly after the surgical region was irrigated with normal saline to ensure everything went well (Figure 1 and Figure 2).

**Figure 3:**
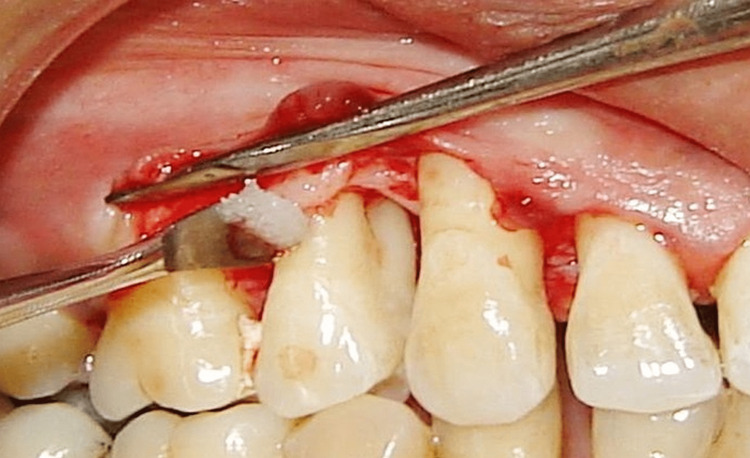
rhBMP-2 along with bone graft placed rhBMP-2: recombinant human bone morphogenetic protein-2

Only OFD was performed at the control site. The test site received an rhBMP‑2 bone graft substitute. The preparation of rhBMP‑2 was initiated approximately 15 minutes before application. Once the rhBMP-2 was ready, it was placed immediately on the exposed root surface so that the rhBMP‑2 touched the root surface (Figure 3).

Because saliva could contaminate the rhBMP-2 and diminish its efficacy, special care was taken to keep it away from the root surface and the defect site during grafting procedures. The interdental sutures (3-0 black braided silk sutures) used to secure the gingival flaps in both groups allowed soft tissue to adapt to the surgical site optimally. The suggested antibiotics for painkillers and for the surgical period were amoxicillin 500 mg tid for five days and paracetamol 625 mg bid for three days. Patients were instructed to use a chlorhexidine solution diluted to 0.2% for rinsing for four to six weeks after surgery. And, of course, we took note of any untoward experiences. After seven to 10 days, the sutures were taken out. In this study, we measured clinical indicators, such as PI, gingival index, PPD, clinical attachment level, and radiographic intraosseous defects, at baseline, three months, six months, and nine months.

**Figure 4:**
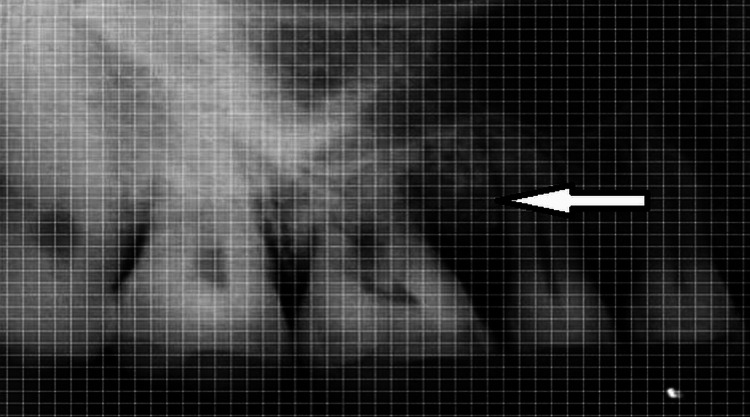
Bone levels after nine months duration in the same region

A statistical analysis was performed on all of the collected clinical parameters using IBM SPSS Statistics for Windows, Version 21.0 (Released 2012; IBM Corp., Armonk, New York, United States). The analysis of variance (ANOVA) and Bonferroni post hoc test were used to examine differences between groups. An unpaired t-test was carried out to evaluate the differences between the two groups.

## Results

PPD, gingival index, and PI were all significantly reduced after periodontal treatment in both groups. Between the two get-togethers, there was a significant improvement in the quality of the connections made. The mean AC/BD was compared between the control and experimental groups at baseline, three months, six months, nine months, baseline-three months, baseline-six months, and baseline-nine months using the unpaired t-test. At both six and nine months, the control group had a significantly higher mean AC/BD. Average AC/BD levels in the experimental group were greater than those in the control group at six and nine months (Table 1).

**Table 1:** Distance from base of the defect to alveolar crest AC: alveolar crest; BD: the base of the defect

	Test		Control		Mean difference	t-test value	p-value
AC/BD	Mean	Std. deviation	Mean	Std. deviation			
Baseline	4.07	0.83	3.94	0.65	0.13	1.289	0.130
Three months	3.50	0.58	3.12	0.82	0.38	-0.265	0.193
Six months	2.55	0.80	2.00	0.88	0.55	2.118	0.048
Nine months	1.15	0.27	1.68	0.27	-0.53	2.360	0.043
Baseline-three months	0.57	0.54	0.82	0.68	-0.25	1.791	0.147
Baseline-six months	1.52	0.99	1.94	0.85	-0.42	2.495	0.027
Baseline-nine months	2.92	0.78	2.26	0.74	0.66	2.699	0.017

The mean CEJ/BD was compared between the two groups at baseline, three months, six months, nine months, baseline-three months, baseline-six months, baseline-nine months, three to six months, three to nine months, and six to nine months using an unpaired t-test. At six and nine months, the mean CEJ/BD of the control group was higher than that of the experimental group. The test group had higher mean CEJ/BD at baseline-six months, baseline-nine months, and six to nine months than the control group did at all three time points (Table 2).

**Table 2:** Distance from cemento enamel junction to base of the defects CEJ: cementoenamel junction; BD: base of the defect

	Test		Control		Mean difference	t-test value	p-value
CEJ/BD	Mean	Std. deviation	Mean	Std. deviation			
Baseline	5.93	1.00	5.86	0.83	0.07	1.858	0.175
Three months	4.89	0.63	5.06	0.77	-0.17	-0.268	0.791
Six months	1.94	0.83	2.50	1.16	-0.56	2.128	0.046
Nine months	1.22	0.27	2.07	0.27	-0.85	2.370	0.045
Baseline-three months	1.04	0.77	0.80	0.68	0.24	1.813	0.165
Baseline-six months	3.99	1.34	3.36	1.31	0.63	2.513	0.030
Baseline-nine months	4.71	0.95	3.79	0.97	0.92	2.711	0.019

The unpaired t-test was used to compare the mean CEJ/AC between the two groups at baseline, three months, six months, nine months, baseline-three months, baseline-six months, baseline-nine months, three to six months, three to nine months, and six to nine months. The mean CEJ/AC in the control group was higher at six and nine months. When comparing the test group to the control group, the test group had a substantially higher mean CEJ/AC at six months, nine months, and six to nine months (Table 3).

**Table 3:** Distance from cemento enamel junction to base of the defect CEJ: cemento enamel junction; BD: base of deflect

	Test		Control		Mean difference	t-test value	p-value
CEJ/AC	Mean	Std. deviation	Mean	Std. deviation			
Baseline	1.81	0.43	1.86	0.36	-0.04	-0.478	0.637
Three months	1.75	0.27	1.81	0.36	-0.06	0.593	0.558
Six months	1.14	0.27	1.53	0.43	-0.39	2.138	0.047
Nine months	0.74	0.00	1.24	0.00	-0.50	2.380	0.048
Baseline-three months	0.06	0.53	0.04	0.00	0.02	1.835	0.183
Baseline-six months	0.67	0.61	0.33	0.50	0.34	2.531	0.033
Baseline-nine months	1.07	0.43	0.62	0.36	0.46	2.723	0.021

Defects were filled to a considerable degree in both groups radiographically; however, the test group’s defect fill was noticeably better than the control group’s. There was evidence that both groups might promote periodontal healing. No immunological or antigenic responses were seen in any of the patients given rhBMP-2, suggesting that it is safe for use.

## Discussion

The use of molecular and cellular biology in the field of periodontal regenerative medicine has led to a deeper understanding of the roles growth factors play and how they interact with the extracellular matrix and cells. When attachment apparatus is lost because of periodontal disease, regeneration of the periodontium to a pre-diseased form is attempted.

In contrast to the gold standard of autogenous bone grafts, there are various investigated and commercially accessible bone grafting replacements on the market. Maximum numbers of essential bone cells are present in autografted bone, making it suitable for all three stages of bone graft development (osteogenesis, osteoinduction, and osteoconduction) [3]. However, there are many challenges associated with autogenous bone grafts, such as limited availability, ease of procurement, the possible need for general anesthesia, hospitalization, and increased morbidity because of the second site of surgery [4,5]. Allografts and xenografts have to some extent solved issues such as limited availability, but the major drawbacks of immunogenicity and possible transmission of HIV remain [6]. Studies have indicated that the outcomes of xenograft application in augmentation are as effective as those of autogenous grafts [7,8]. A lack of revascularization, a lack of creeping substitution, a lack of mineral accretion, and a small number of cells involved in the remodeling process of allograft grafts have all been observed in animal and human model studies, indicating that allograft grafts performed histologically and clinically worse than autografts [3,9]. Alloplasts are readily available and economical bone substitutes, but they lack osteoinductive properties. To overcome these drawbacks of conventional graft substitutes, growth factors such as BMP, PDGF, etc. were incorporated.

BMP is a growth factor that belongs to the family of TGF-β. They were first isolated by Urist in 1965; since then, more than 30 types of BMP have been isolated. BMP-2 has known osteoinductive properties as it induces mesenchymal progenitor cells to differentiate into osteoblasts [10-12]. BMP-2 is a soluble protein that flushes away from the grafted site; therefore, a carrier is required for the controlled spatial release of BMPs [13]. A slow release of BMP is also required for optimal biological properties, as the sudden release of BMP-2 can be associated with localized complications of supra-physiological doses [14]. This has led to a new generation of graft materials known as composite grafts. Composite grafts consist of growth factors loaded into an appropriate carrier. The major challenge to the application of BMP-2 in osseous regenerative surgery was its availability. Owing to its limited availability, low yield, and high cost of processing, the application of BMP-2 composite grafts was not commercially feasible [15]. With the advancement in molecular and genetic engineering, the fabrication of rhBMP-2 was made possible. Recombinant technology spliced and amplified human BMP-2, and quantitative production of rhBMP-2 was made possible [16].

These findings are similar to those of Guimaraes et al. [17]. At six months, there was no statistically significant difference between the test (BMP + GTR) and control groups in PPD reduction and CAL improvement in a study of 15 patients with 10 pairs of intrabony defects treated with pooled BMP + GTR. The drawback of this study is that it was done over a six-month period without a radiographic assessment. However, this study was done over a nine-month period and evaluated both clinically and radiographically. The amount of rhBMP‑2 used was 3-12mg, which was adequate to bring about clinically significant changes in the test sites. The PD depth reduction and CAL improvement showed no significant difference at six months between the BMP (test) and OFD (control) groups.

However, at nine months, there was a significant reduction in PD and CAL improvement in BMP-treated sites than in the control sites, and the study done by Vandana et al. [18], in which 32 patients with intraosseous abnormalities treated with rhBMP-2 computer-generated tables were used to randomly assign locations to either the control or the experimental groups. Sites treated with OFD served as the control group, whereas sites treated with OFD and rhBMP-2 showed that neither PPD nor CAL were significantly different between the experimental (BMP) and the control groups (OFD) at baseline. From baseline to six and nine months, PD decreased considerably in both groups. From pre- to post-test at six and nine months, there was a statistically significant increase in CAL across all groups. At six months, there was no statistically significant difference in CAL improvement between the groups. At six months (p = 0.002) and nine months (p = 0.001), there was significantly more bone fill in the BMP group than in the OFD group. At six and nine months, the BMP group significantly outperformed the control group in terms of the percentage of original defects resolved.

The findings of this study suggest that the use of rhBMP‑2 provided PPD reduction, CAL improvement, and significant radiographic enhancement of the original defect resolution of intraosseous defects at nine months. No adverse reactions were found locally or systemically in the groups.

The study limitations are regarding the sample size, which is small, the fact that the bony defects were not surgically reopened to check bone fill, and the fact that histological analysis was not performed.

## Conclusions

Evidence from this research reveals that rhBMP-2 significantly reduces plaque count, gingival filing, pocket depth, clinical connection level, and radiographic bone fill. Open flap debridement with rhBMP-2 and alloplastic bone grafts shows better reduction than open flap debridement with alloplastic bone grafts in radiographic defect fill.
